# *ACE* and *ACTN3* Gene Polymorphisms and Genetic Traits of Rowing Athletes in the Northern Han Chinese Population

**DOI:** 10.3389/fgene.2021.736876

**Published:** 2021-10-14

**Authors:** Qi Wei

**Affiliations:** ^1^ Key Laboratory of General Administration of Sport of China, Wuhan, China; ^2^ Hubei Institute of Sports Science, Wuhan, China

**Keywords:** angiotensin converting enzyme, α-actin-3, single nucleotide polymorphism, trait variables, genetic model

## Abstract

This investigation aimed to explore the effects of *ACE* I/D and *ACTN3* R577X gene polymorphisms on specific quantitative variables, including height, weight, arm span, biacromial breadth, forced vital capacity (FVC), FVC/weight, maximal oxygen uptake (VO_2_max), prone bench pull (PBP), loaded barbell squat (LBS), and 3,000-m run, in 243 Chinese rowing athletes. The *ACE* and *ACTN3* genotypes were obtained for each athlete *via* polymerase chain reaction on saliva samples, and the genotype frequency was analyzed. The *ACE* genotype frequency of rowing athletes were 45.8% II, 42.2% ID, and 12% DD for males and 33.6% II, 48% ID, and 18.4% DD for females. There were significant differences in weight in male athletes, PBP in female athletes, and *ACE* genotypes. A linear regression analysis using PBP and LBS as different dependent variables and *ACE* genotypes as independent variables based on the *ACE* I allele additive genetic effect showed a statistical significance in female athletes (*p* < 0.05). There was a significant difference in the distribution of the three genotypes among male athletes (36.7% XX, 38.5% RX, and 24.8% RR, *χ*2 = 5.191, df = 2, *p* = 0.022 < 0.05). There were no significant differences in the distribution of the three genotypes among female athletes (23.8% XX, 47.8% RX, 28.4% RR, *χ* ^2^ = 0.24, df = 2, *p* = 0.619 > 0.05). The *ACTN3* gene polymorphism of male rowing athletes was dominated by the *ACTN3* 577X allele. There were significant differences in the *χ* ^2^ test between groups of male athletes. The *ACTN3* R577 allele was dominant in female athletes. There were significant differences between PBP and FVC/body weight and *ACTN3* genotypes in male athletes by ANOVA, respectively (*p* < 0.05). A linear regression analysis using FVC and FVC/body weight as dependent variables and *ACTN3* genotypes as independent variables based on the *ACTN3* 577X allele recessive genetic effect showed statistical significance in male athletes (*p* < 0.05). These results suggested that *ACE* and *ACTN3* gene polymorphisms may be used as biomarkers of genetic traits in Chinese rowing athletes.

## Introduction

Human physical performance is widely accepted as individual characteristics dependent on interactions between genes and environment (Bouchard, 2011). Sports phenotypes, such as endurance, explosive power, muscle fiber type and proportion, and flexibility, are highly influenced by genetic and epigenetic factors (Zempo et al., 2017; Miyamoto-Mikami et al., 2018). These reviews showed the heritability of body height, body mass index, VO_2_max adjusted for body weight, isometric grip strength, other isometric strength, isotonic strength, isokinetic strength, jumping ability, and other power measurements by meta-analysis 0.87–0.93 (Silventoinen et al., 2003), 0.47–0.9 (Elks et al., 2012), 0.56 (Miyamoto-Mikami et al., 2018), 0.56, 0.49, 0.49, 0.49, 0.55, and 0.51, respectively (Zempo et al., 2017). Environmental effects begin as early as preconception *via* gametic imprinting and continue after conception during the growth and development period (Bouchard, 2011).

Studies have suggested that genes are partially responsible for determining the anthropometric, physical, and physiological traits needed to achieve athletic performance (Pickering, 2019). Single-nucleotide polymorphisms (SNPs) are genetic sequence variations related to the expression variation of key genes regulating the physiological process of exercise (Boulygina, 2020). With the continuous development of genetic technology, it has become easier to further explore the genetic basis of excellent sports performance and find that SNPs and other genetic variations may directly or indirectly influence the athletic performance of aerobic exercise ability and other physiological characteristics, such as muscle strength and speed. Many SNPs associated with high heritability of phenotypes related to athlete status have been identified in the last 2 decades (Maciejewska-Skrendo et al., 2019; Semenova et al., 2019; Valeeva et al., 2019). The *ACE* I/D and *ACTN3* R577X polymorphisms have been intensely investigated with athletic performance in endurance- and power-oriented events (Jacob et al., 2018).

The *ACE* gene is a 21-kb single-copy gene located on 17q23 that encodes angiotensin-converting enzyme (ACE), which regulates human circulatory homeostasis, skeletal muscle growth, and cardiovascular functions; its I/D polymorphism denotes either an insertion or deletion of a 287-bp Alu repeat sequence at intron 16 (Pescatello et al., 2019). In 1998, the *ACE* I allele was found to be significant in elite British mountaineers (Montgomery et al., 1998). The proportion of Australian elite rowers carrying the *ACE* I allele was higher than that of the control group. The II genotype tends to reduce cardiac afterload during exercise and can effectively couple the ventricle and blood vessels to improve exercise endurance (Gayagay et al., 1998). Studies have speculated that the *ACE* I allele may decrease ACE enzyme activity to enhance human endurance performance (Ma et al., 2013; Pescatello et al., 2019). The D allele is associated with increased muscle volume-related baseline and rapid-twitch muscle fiber proportions with greater strength performance (Eider et al., 2013).

The *ACTN3* gene is located on chromosome 11, and its polymorphism is caused by the C-T polymorphism mutation at the R577X site, which produces a stop codon, resulting in the change of the amino acid at 577 from arginine (577 R) to a stop code (577X). Muscle contraction strength and speed are required at high levels of activity, and subjects carrying the 577X allele encode an early termination of actin3, resulting in muscle loss that affects muscle performance. Approximately 18% of the world population (approximately 1.5 billion people) has the XX genotype and deficiency of α-actinin-3 (ACTN3) without causing any significant muscle disease (Yang et al., 2003). However, some research found that XX homozygosity leading to ACTN3 deficiency can adversely impact sports performance through muscle type and energy metabolism (Papadimitriou et al., 2008; Cięszczyk et al., 2011; Mikami et al., 2014).

Rowing is competed over a 2,000-m track, requiring the rowers to exhibit extreme physiological power and endurance, technical proficiency, and environmental characteristics (Keenan et al., 2018). Studies have found that the performance of the rowers is related to individual anthropometric variables, such as height, weight, length of legs and body span, and muscular strength in the trunk and upper and lower limbs (Majumdar et al., 2017; Keenan et al., 2018; Maciejewski et al., 2019; Penichet-Tomas et al., 2019). Previous studies are limited to simulated case–control evaluations of *ACE* and *ACTN3* polymorphism designs based on exercise state without quantitative traits of athlete performance. This study is the first to explore the contributions of *ACE* and *ACTN3* gene polymorphisms and the effects of different alleles on sport performance-related phenotypic indicators of Asian athletes, including anthropometric, physical, and strength trait variables of Chinese elite rowing athletes, using one-way ANOVA and linear regression analysis based on two genetic models. We hope to determine the genetic effects and contributions of *ACE* and *ACTN3* gene polymorphisms to the phenotypic indexes related to Chinese rowing athletic ability, which may be conducive to cultivating outstanding athletes.

## Methods

### Participants

We recruited 243 open-category elite rowers: 109 male athletes with an average age of 21.73 ± 2.32 years and training duration of 7.9 ± 1.8 years and 134 female athletes with an average age of 20.58 ± 1.24 years and training duration of 7.5 ± 1.4 years. All subjects were Han Chinese from five provinces (Henan, Shangdong, Hubei, Liaoning, and Jiangsu) who participated in the 2020 National Rowing Championship, including 36 international-level athletes (21 male and 15 female), 54 national-level athletes (24 male and 30 female), and 153 national second-level athletes (64 male and 89 female). The Sports Medicine Committee of the Hubei Sports Science Society Review Board approved the project, and written informed consent was obtained from all participants before testing. The guidelines for Strengthening the Reporting of Genetic Association Studies (Little et al., 2009), an extension of the Strengthening the Reporting of Observational Studies in Epidemiology statement, were followed to report the results of this study.

### Genotyping

Human genomic DNA was isolated from 2 ml of saliva sample collected in Oragene DNA OG-500 collection tubes (DNA Genotek, Canada) and stored at room temperature *via* a DNA extraction and purification kit (DNA Genotek prepIT-L2P, Canada). The primer was synthesized by Wuhan Gene Create Biological Engineering Co., and the amplification primer was also a sequencing primer. A 50-μl reaction system was used for polymerase chain reaction (PCR) amplification, which consisted of 50–80 μg/ml template DNA 2 μl, 10× KoD Buffer 5 μl (TOYOBO, Tokyo), 10 mmol/L forward primer and reverse primer 1.5 μl each, 2 mmol/L dNTPs 4 μl, 25 mM MgSO4 1 μl, KoD-plus amplification enzyme 1 μl (TOYOBO, Tokyo), and was supplemented with deionized water to 50 μl. PCR amplifications were performed by Applied Biosystems PCR (Thermo Fisher Scientific, United States). The primers and PCR conditions for the polymorphism analysis of the *ACE* and *ACTN3* genes are shown in Table 1.

**TABLE 1:** Primers and PCR conditions for polymorphism of *ACE* and *ACTN3* genes.

		*ACE* I/D, rs1799752		*ACTN3* R577X*,* rs1815739	
SNP, primers sequence(5' -3' )		F:5’ CTG GAG ACC ACT CCC ATC CTT TCT 3’		F: 5′-CTG TTG CCT GTG GTA AGT GGG-3′	
		R:5’ GAT GTG GCC ATC ACA TTC GTC AGA 3’		R:5′-TGG TCA CAG TAT GCA GGA GGG-3′	
Protocol(30 cycles)	Predenaturation	95°C	3 min	95°C	3 min
	Denaturation	95°C	30 s	95°C	30 s
	Annealing	60°C	30 s	68°C	30 s
	Extension	72°C	90 s	68°C	90 s
	extension(The final cycle)	72°C	8 min	68°C	8 min

The PCR products of the *ACE* gene were separated *via* 6% polyacrylamide gel electrophoresis and confirmed as follows: 190-bp fragment for DD genotype, 490-bp fragment for II genotype, and 490- and 190-bp fragments for ID genotype. The PCR products digested by the DdeI restriction enzyme (Promega) of each sample were detected as follows: 108-, 97-, and 86-bp fragments for the 577X allele; 205 and 86 bp for the 577 R allele; and sequenced by an ABI 3730 DNA Analyzer (Thermo Fisher Scientific, United States) to identify the *ACTN3* genotypes as reported by Eynon *et al.* (2009).

### Measurement of Anthropometric Trait Variables

The athletes were required to wear sports attire, and measurements of their height, arm span, and biacromial breadth were performed using a large anthropometer (Anthroscan 3D VITUS, Human Solution, Germany). After removing their shoes at 7:00 in the morning, the height of the athletes was measured in an upright position, from the vertex to the floor and from the akropodion to dactylion, as morphological and technical parameters. Bisacromial breadth is usually considered the distance between the two acromial processes. Arm span was measured with the athletes standing straight while the arms were maximally stretched horizontally (making a 90° angle with the trunk). The distance between the tips of the middle fingers was then measured by an anthropometer.

Then, the athletes were measured by a body composition analyzer (X-Scan Plus II, Jawon, Korea) to obtain their weight. All of the measurements were repeated three times by the same observer, and the average of the measurements was taken as the final value.

### Measurement of Physical and Strength Trait Variables

Spirometry was performed using the Chest Microspiro HI-501 vital capacity instrument (CHEST M.I. Inc, Tokyo, Japan). After maximal inhalation, the athletes sealed their lips around the mouthpiece and exhaled as hard and as fast as possible, and the FVC values were displayed digitally. Each athlete completed two tests, with an interval of 30 s, and the highest value was recorded. Finally, the test data were printed.

The VO_2_max test was conducted according to previously reported procedures (Izquierdo-gabarren et al., 2010). The experimental equipment used was the German lung function tester MAX II connected to the German H/P/PULS platform.

Prone bench pull (PBP) and loaded barbell squat (LBS) were performed with the use of standard free-weight equipment (Salter, Madrid, Spain) according to the test methods (Riboli et al., 2021).

### Statistical Analysis

The data in this study were statistically analyzed using SPSS 21.0 for Windows software. The frequency distribution of the *ACE* and *ACTN3* genotypes was verified by the Hardy–Weinberg equilibrium (HWE) law. We converted the 3,000-m run times to seconds and compared the height, weight, arm span, biacromial breadth, VO_2_max, FVC, FVC/body weight, PBP, LBS, and 3,000-m run between the *ACTN3* R577X or *ACE* I/D genotypes using one-way ANOVA. Considering that rowing performance is mainly based on aerobic stamina, we selected two kinds of genetic models to calculate the genetic effects of the advantage of the *ACE* I allele and the 577X allele. One was the additive model (*i*.*e*., the R/R = 0, R/X = 1, X/X = 2, or D/D = 0, I/D = 1, I/I = 2); the other was the recessive model (R/R = R/X = 0, X/X = 1, Or D/D = I/D = 0, I/I = 1) (Papadimitriou et al., 2018). A simple linear regression using the anthropometric, physical, and strength traits as the dependent variables and the genotypes of the two genetic models as independent variables were then applied, and the significance level was set at *p* < 0.05. The male and female athletes were analyzed separately.

Considering the effect of genetic structure in athletes with different geographical locations, we searched public databases from the NCBI Allele Frequency Aggregator (ALFA), Genome Aggregation database (gnomAD), 1,000 Genomes Project (1KGP), and China Metabolic Analytics Project (ChinaMAP) (Cao et al., 2020) for the different population gene allele frequency data for *ACE* I/D (rs1799752) and *ACTN3* R577X (rs1815739) loci.

## Results

### Distributions of the *ACE* I/D Polymorphism and the *ACTN3* R577X Polymorphism in Rowing Athletes

**FIGURE 1:**
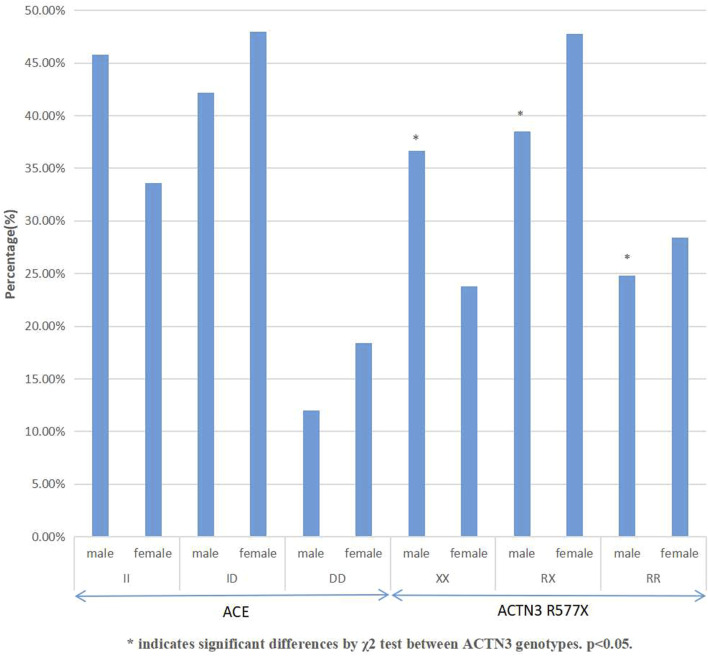
Distribution of *ACE* and *ACTN3* gene polymorphisms in elite Chinese rowers.

The genotype and allele frequency of the *ACE* I/D and the *ACTN3* R577X polymorphisms of rowing athletes in this study were tested by the HWE and *χ* ^2^ test, indicating that the subjects selected in this study were representative of the population (*p* > 0.05). In Figure 1, the frequency of the *ACE* I allele of male athletes was 66.9%, while the frequency of the D allele was 33.1%. The genotypes of male athletes were 45.8% II, 42.2% ID, and 12% DD. There was no significant difference in the distribution of the three genotypes among male athletes (*χ* ^2^ = 0.23, df = 2, *p* = 0.63 > 0.05). The *ACE* I allele frequency of female athletes was 57.5%, and the D allele frequency of female athletes was 42.5%. The genotypes of female athletes were 33.6% II, 48% ID, and 18.4% DD, with no significant differences among the three genotypes (*χ* ^2^ = 0.07, df = 2, *p* = 0.79 > 0.05).

The frequency of the *ACTN3* 577X allele of male athletes was 55.9%, while the frequency of the *ACTN3* R577 allele was 44.1%. The genotypes of male athletes were 36.7% XX, 38.5% RX, and 24.8% RR. There was a significant difference in the distribution of the three genotypes among male athletes (*χ* ^2^ = 5.191, df = 2, *p* = 0.022 < 0.05). The 577X allele frequency of female athletes was 47.8%, and the 577 R allele frequency of female athletes was 52.2%. The genotypes of female athletes were 23.8% XX, 47.8% RX, and 28.4% RR, with insignificant differences among the three genotypes (*χ* ^2^ = 0.24, df = 2, *p* = 0.619 > 0.05).

**FIGURE 2:**
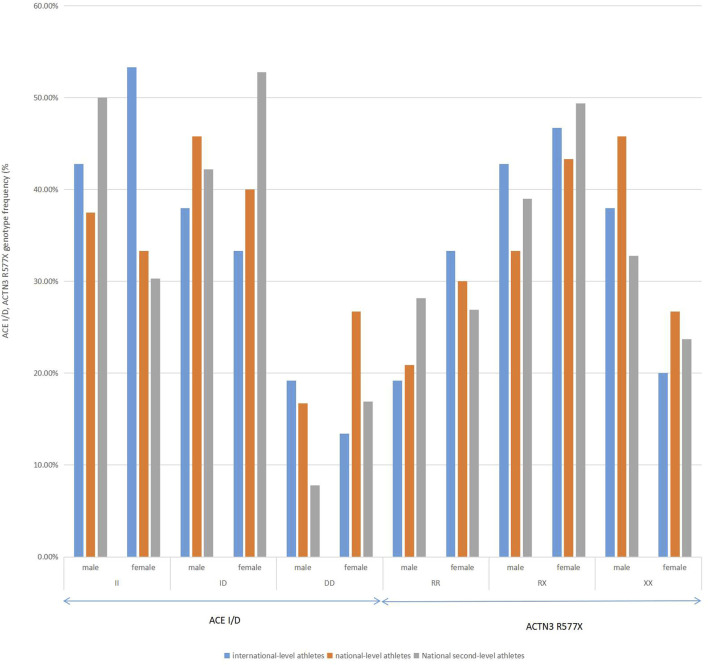
Distribution of *ACTN3* gene polymorphism in male international-level athletes, national-level athletes, and national second-level athletes of rowing in China.

Subgroup comparisons among different levels of rowing athletes. including international-level athletes, national-level athletes, and national second-level athletes, were performed by gender in Figure 2. There were no significant differences in the distributions of the *ACE* genotype among the subgroups of male and female athletes (*χ* ^2^ = 0.532, df = 2, *p* = 0.323 > 0.05; *χ* ^2^ = 0.583, df = 2, *p* = 0.317 > 0.05). There was a significant difference among the three genotypes of *ACTN3* R577X in the subgroup of male athletes (*χ* ^2^ = 2, df = 2, *p* = 0.007 < 0.01). There were no significant differences among the three genotypes of *ACTN3* R577X in the subgroup of female athletes (*χ* ^2^ = 0.471, df = 2, *p* = 0.411 > 0.05).

**FIGURE 3:**
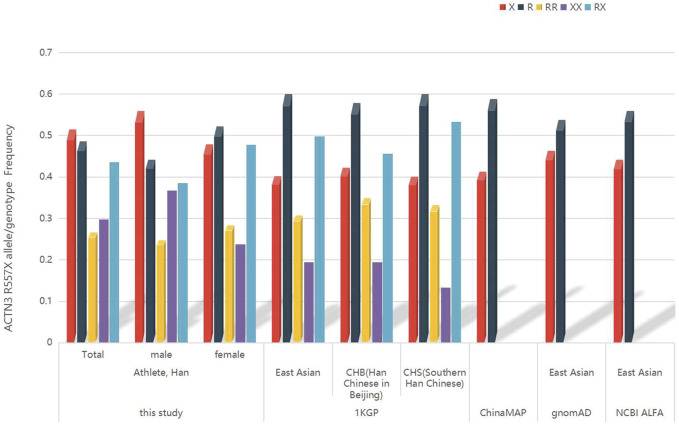
The frequency of *ACTN3* R577X allele/genotype in rowing athletes in China, Chinese population from the ChinaMAP, East Asian, Han Chinese in Beijing, and Southern Han Chinese populations from 1KGP, East Asian population from gnomAD, and NCBI ALFA.

In Figure 3, the frequency of the *ACTN3* 577X allele among all athletes in this study was 51.44%, which was higher than the allele frequency among the total Chinese population in ChinaMAP, the East Asian, Han Chinese in Beijing (CHB), and Southern Han Chinese (CHS) populations from 1KGP, and the East Asian populations from gnomAD and NCBI ALFA. The frequency of the *ACTN3* R577 allele among all athletes in this study was 48.56%.

### Analysis of *ACE/ACTN3* Polymorphisms and Trait Variables of Rowers

The anthropometric, physical, and strength trait variables related to rowing sports, according to the *ACE* and *ACTN3* genotype and distribution, are shown in Table 2 and Table 3. There was a significant difference between weight and *ACE* genotypes, FVC/body weight, and *ACTN3* genotypes in male athletes by ANOVA, respectively (*p* < 0.05). The linear regression analysis of FVC and FVC/body weight as different dependent variables and *ACTN3* genotypes as independent variables based on the 577X allele recessive genetic effect was statistically significant in male athletes (*p* < 0.05).

**TABLE 2:** The ACE I/D genotype and distribution in rowing athletes showing the related anthropometric, physical and strength-trait variables.

	ACE (male)						ACE (female)					
	Genotypes			ANOVA	The linear regression		Genotypes			ANOVA	The linear regression	
	II	ID	DD	*p*	Additive	Recessive	II	ID	DD	*p*	Additive	Recessive
	45.8%	42.2%	12%		(DD= 0, ID=1, II =2)	(DD= ID=0, II =1)	33.6%	48%	18.4%		(DD= 0, ID=1, II =2)	(DD= ID=0, II =1)
	(N=50)	(N=46)	(N=13)				(N=45)	(N=64)	(N=25)			
Height, cm	191.61±3.29	189.08±3.75	193±1.22	0.059	0.311	0.992	177.58±6.88	176.16±6.46	173.2±3.23	0.671	0.37	0.509
Weight, kg	84.81±10.87	76.74±11.21	90.84±5.94	0.040^*^	0.206	0.842	65.52±7.43	69.22±10.91	62.46±9.28	0.58	0.8	0.755
Arm span, cm	195.6±2.12	189.67±3.21	191.82±1.58	0.067	0.359	0.888	180.33±6.74	177.78±5.01	173.2±3.95	0.404	0.173	0.334
Biacromial breadth, cm	42.67±1.21	42±1	42.8±2.04	0.766	0.911	0.841	39.16±1.89	39.75±1.54	38.5±1.32	0.551	0.617	0.879
VO_2_max, L min^−1^ kg^−1^	4.81±0.58	4.21±0.1	5.17±0.34	0.428	0.639	0.438	3.05±0.14	3.68±0.15	3.22±0.8	0.215	0.692	0.214
FVC, ml	6012±983	5265.5±1117	5728±751	0.609	0.872	0.745	3733±394	4083±643	3673±342	0.577	0.973	0.549
FVC/body weight, ml kg^−1^	68.83±9.72	60.25±24.39	63.85±5.67	0.598	0.523	0.351	59.6±8.62	61.05±5.65	63.6±8.33	0.777	0.474	0.619
PBP, kg	84±9.69	86±3.6	84±5.47	0.92	0.986	0.85	76.6±14.43	69±12.75	50±18	0.115	0.043^#^	0.222
LBS, kg	118±11.04	128.33±2.88	124±8.94	0.296	0.293	0.145	113.3±11.54	111.16±14.4	68.3±24.6	0.0092^&^	0.018^#^	0.345
3,000-m run time,s	637.17±34.43	576.67±5.77	526.67±59.35	0.068	0.745	0.232	742.3±53.46	739.16±34.98	741.67±73.22	0.995	0.987	0.965

Notes: * indicates that a significant difference between the trait variables and ACE three genotypes in male athletes (*p* < 0.05); & indicates that a significant difference between the trait variables and ACE three genotypes in female athletes (*p* < 0.05); # indicates that the linear regression analysis of trait variables and ACE genotypes based on ACE I dominant genetic effect has very significant statistical significance in female athletes (*p* < 0.05).

**TABLE 3:** The ACTN3 R577X genotype and distribution in rowing athletes showing the related anthropometric, physical and strength-trait variables.

	ACTN3 (male)						ACTN3 (female)					
	Genotypes			ANOVA	The linear regression		Genotypes			ANOVA	The linear regression	
	RR	RX	XX	*p*	Additive	Recessive	RR	RX	XX	*p*	Additive	Recessive
	24.8%	38.5%	36.7%		(RR= 0, RX=1,XX=2)	(RR=RX=0, XX =1)	28.4%	47.8%	23.8%		(RR= 0, RX=1,XX=2)	(RR=RX=0, XX =1)
	(N=27)	(N=42)	(N=40)				(N=38)	(N=64)	(N=32)			
Height, cm	189.63±4.89	192±2.89	191.83±2.59	0.494	0.425	0.232	172.46±2.92	177.56±6.17	175.22±6.15	0.41	0.698	0.716
Weight, kg	75.8±12.27	86.9±9.39	87±10.7	0.226	0.188	0.081	61.06±6.8	70.28±9.76	63.52±8.45	0.269	0.884	0.455
Arm span, cm	191.55±0.49	193.1±2.7	190.3±3.84	0.436	0.566	0.803	176±6.4	177.1±5.85	178.7 ±6.4	0.892	0.622	0.642
Biacromial breadth, cm	42.5±0.7	42.57±1.71	42±1.17	0.7787	0.658	0.944	39.12±1.54	39.75±1.54	38.75±2.47	0.805	0.915	0.804
VO_2_max, L min^−1^ kg^−1^	4.21±0.58	5.02±0.47	4.86±0.6	0.372	0.906	0.234	3.05±0.14	3.43±0.63	3.63±0.21	0.436	0.193	0.214
FVC, ml	4585.5±232	5786±691	5538±1015	0.149	0.092	0.049^%^	3726±327	4083±643	3099±342	0.135	0.819	0.54
FVC/body weight, ml kg^−1^	44.4±27.7	68.4±9.56	65.3±6.17	0.047^%^	0.113	0.012^€^	61.5±7.61	61.05±5.65	58.4±1.13	0.776	0.778	0.919
PBP, kg	86.5±4.94	87.71±6.44	79±5.47	0.079	0.071	0.666	71.5±15.67	60.5±12.75	72.5±17.67	0.543	0.839	0.459
LBS, kg	127.5±3.53	126.14±7.58	115±10	0.087	0.045^	0.432	108±14.23	92.5±32.2	112.5±10.6	0.53	0.937	0.517
3,000-m run, s	575±7.07	637±52.2	516.5±25.5	0.095	0.669	0.08	746±43.84	746.67±37.67	728.75±65.13	0.843	0.614	0.548

Notes: % indicates that a significant difference between the trait variables and ACTN3 three genotypes in male athletes (*p* < 0.05); ^ indicates that the linear regression analysis of trait variables and ACTN3 genotypes based on ACTN3 X dominant genetic effect has statistical significance in male athletes (*p* < 0.05); € indicates that the linear regression analysis of trait variables and ACTN3 genotypes based on ACTN3 R dominant genetic effect has statistical significance in male athletes (*p* < 0.05).

ANOVA revealed a significant difference between PBP and *ACE* genotypes in female athletes (*p* < 0.01). The linear regression analysis of the PBP and the LBS as different dependent variables and *ACE* genotypes as independent variables based on the *ACE* I allele additive genetic effect showed a statistical significance in female athletes (*p* < 0.05). The trait variables of female athletes and *ACTN3* genotypes had no statistical significance in the linear regression analysis (*p* > 0.05).

## Discussion

Olympic rowing is a typical strength/power endurance sport in which physical fitness, strength, rowing techniques, and tactics influence rower success. The anthropometric length or breadth of the human body is almost entirely genetically decided and can hardly be altered within the range of training periodization (Almeida-Neto et al., 2020). Numerous studies found that 2,000-m rowing ergometer performance was predicted by body mass, VO_2_max (Almeida-Neto et al., 2020), age, height, weight, and body fat percentage (Majumdar et al., 2017).

Through twin studies, genetic factors have been found to have an important impact on muscle strength, flexibility, and balance. The heritability of standing long jump is 62%, grip strength 63%, balance 35%, and flexibility 50% (Schutte et al., 2016). Many SNPs have been found to be related to the physiological characteristics of elite athletes, such as muscle power, speed, and aerobic capacity. Height and body mass are highly heritable and contribute to performance.

Studies have consistently provided more associations between genotype II and endurance capability (Ma et al., 2013). The *ACE* I allele is highly expressed in British climbers (Montgomery et al., 1998), Australian athletes (Gayagay et al., 1998), Polish male rowers (Jastrzębski et al., 2014), Russian rowing athletes (Ahmetov et al., 2008), Chinese female soccer athletes (Qi, 2021), and Tunisian athletes (Znazen et al., 2015). Another study found that the proportion of endurance athletes carrying the *ACE* I allele was not significant (Papadimitriou et al., 2018). Aerobic endurance refers to the ability of the body to maintain aerobic exercise. VO_2_max is a high heritability index for quantifying aerobic endurance. The heritability of VO_2_max can reach 80–90%, and it can be increased by 20–25% through exercise training. The cardiovascular system of the body delivers oxygen to the muscles during exercise so that the muscles can use oxygen to exercise. ACE promotes the synthesis of aldosterone and the degradation of angiodilators through the synthesis of angiotensin II and plays a tonic regulatory role in circulatory homeostasis. In this study, the athletes were all of Han nationality, and there were no differences between *ACE* genotypes. The proportion of individuals carrying the *ACE* I allele was higher than that of individuals carrying the D allele. The majority of male athletes had the II genotype, and weight was significantly different among the three *ACE* genotypes. We constructed two genetic models to calculate the *ACE* I allele genetic effects on trait variables of the linear regression analysis. The weight and other trait variables of the male athletes had no relationship with the *ACE* genotype (*p* > 0.05) in the linear regression, which suggested that the *ACE* I allele may have no genetic effect on weight in either homozygotes or heterozygotes.

Research suggests that mononuclear cells and ACE enzyme activity of the heart of the II genotype, compared with the DD genotype, can strengthen the myocardial contraction and cardiac output and by adjusting the level of bradykinin substrates and affect the growth of skeletal muscle energy metabolism. A high expression of the *ACE* I allele can enhance muscle oxygen absorption (Tsianos et al., 2004). Excessive body fat may reduce human body oxygen consumption and affect aerobic capacity. The *ACE* gene was found to be associated with slow-twitch type I muscle fiber (Papadimitriou et al., 2016). Higher oxygen availability and nutrient delivery for muscle fibers in contraction decrease the ACE serum levels and activity (Gunel et al., 2014). The majority of female athletes in this study had the ID genotype, with LBS revealing a very significant difference between *ACE* genotypes by ANOVA (*p* < 0.01). With the *ACE* I allele additive genetic model, it was further found that the PBP and LBS of female athletes showed significant linear regression with the *ACE* genotype (*p* < 0.05 and *p* < 0.01, respectively). PBP and LBS were both associated with muscle strength. The Caucasus power projects excellent athletic sports ability associated with the *ACE* D allele (Kim et al., 2010; Scott et al., 2010). The *ACE* D allele may improve blood ACE activity and the content of angiotensin II to transfer higher muscle strength (Charbonneau et al., 2008). Based on the representation of the *ACE* D allele of female athletes in this study, it is speculated that the effect of DD homozygotes on the muscle type of female athletes is more obvious.

The highest prevalence of the *ACTN3* gene polymorphism was the heterozygous RX genotype in male and female rowing athletes. Male rowing athletes in this study mainly carried the 577X allele, and the proportion of male athletes was 38.5% RX, which was significantly different from 36.7% XX and 24.8% RR among the groups (*p* < 0.05). The same finding was found in Australian endurance athletes (Yang et al., 2003) and Israeli top-level long-distance runners (Jastrzebski et al., 2014). The 577X allele with a high proportion of slow muscle fibers is associated with endurance events (Papadimitriou et al., 2018). In particular, studies have reported that the 577X allele is underrepresented in Russian male rowers (Ahmetov et al., 2008; Cięszczyk et al., 2011), Chinese male endurance athletes (Shang et al., 2010), and Polish rowers (Cieszczyka et al., 2012; Jastrzebski et al., 2014). There was a significant difference among the three genotypes of *ACTN3* R577X in the subgroup of male athletes. The *ACTN3* XX genotype frequency in Chinese female endurance athletes is significantly linearly increasing among average, sub-elite, elite, and highly elite athletes (Shang et al., 2010). On the contrary (Cieszczyka Pawel et al., 2012), analyzed that the genotype distribution and allele frequency between the elite and non-elite were not significantly different (*p* = 0.82 and *p* = 0.56, respectively).

The athletes in this study were recruited from the Han Chinese population in the northern and central provinces of China. The Han Chinese, an ethnic group native to China, is the largest ethnic group in the world, distributed in East Asia, Southeast Asia, and other parts of the world (Chen et al., 2019). Paleolithic ancient Tianyuan DNA (Mao et al., 2021) showed the paleolithic–modern genomic continuity in East Asia. In recent years, the Simons Genome Project, 1KGP, Human Genome Diversity Project, and HapMap Project have been conducted to study the genetic diversity and population structure of East Asians (Li et al., 2008; Consortium et al., 2009; Sudmant et al., 2015; Mallick et al., 2016). Genetic admixture with local ethnic groups and substantial genetic diversity within Han Chinese have been reported in previous studies. He *et al.* genotyped 36 Tai-Kadai-speaking Qiongzhong Hlai and 48 Haikou Han individuals at 497,637 SNPs, which revealed that East Asian populations are characterized by a north–south genetic cline (He et al., 2020). Tujia people and central Han Chinese suggested a genetic admixture under language borrowing (He G. L. et al., 2021). Genetic studies based on higher-resolution, genome-wide autosomal and uniparental Y-chromosome and mitochondrial deoxyribonucleic acid SNP data from 599 Northwest Han (Gansu Province) individuals showed increased genetic homogeneity in northwest Han individuals relative to the Mongolian/Turkic/Tungus and Tibetan–Burmese populations in the north (Yao et al., 2021). The 986 previous genome-wide analyses from southernmost, central, and northern modern Han Chinese are consistent with the primary ancestry of modern southeastern coastal Han Chinese originating from northern China (He G. et al., 2021; He G. G. et al., 2021). There was a genetic substructure in Shaanxi Han in terms of north–south-related ancestry corresponding well to latitudes (He G. L. et al., 2021) and great genetic differentiation compared to Guizhou Han (Wang et al., 2021). The ChinaMAP has established a large-scale resource of 10,588 individual deep whole-genome sequencing data and the genetic bases of metabolic traits for the genetic study of East Asians.

Considering the effect of genetic structure with different geographical locations, we searched the *ACTN3* R577X frequency of the East Asian population from public databases. The *ACTN3* 577X allele frequency of all athletes was higher than that of other populations. Furthermore, the frequency of XX homozygote among all athletes was higher than that of East Asian, CHB, and CHS populations from the 1KGP and revealed the endurance ability cline of the athlete population. We speculated that the differences between the athlete population and those described above are because the athlete population was selected based on their athletic performance and influenced by the geographic/genetic diversity of northern and central China. The fine genetic structure of the athletes will be considered in the design of further studies.

Through ANOVA, we found that FVC/body weight was significantly different among the *ACTN3* R577X genotypes (*p* < 0.05), and FVC and FVC/body weight had a statistically significant linear regression with the *ACTN3* genotype based on the 577X allele recessive genetic model (*p* < 0.01). It was speculated that, for male athletes, RR and XX homozygosity had a genetic influence on FVC and FVC/body weight, respectively. α-Actinin-3 is encoded by the *ACTN3* gene, and its expression in glycolytic skeletal muscle contributes to enhancing muscle function and coordinating fast-twitch muscles (Yang et al., 2003). Ortiz et al. (2022) reported that Colombian athletes carrying the RX genotype might express more ACTN3 protein that is involved in the optimization of muscle contraction in fast-twitch fiber. The frequency of the RR genotype was revealed to be higher in male skiers than in the control group, but male skiers with the XX genotype evidently had an increased VO_2_ peak within 5 years (Magi et al., 2016). Pimenta *et al.* (2013) found that VO_2_max in males with the XX genotype was higher than that in males with the RR genotype. In mice with an *ACTN3* gene knockout model, the loss of ACTN3 protein induces the transformation of skeletal muscle fast-twitch fibers to oxidative metabolism, and the increase in actin-2 levels can work as a compensatory mechanism for the loss of ACTN3 protein, which is conducive to endurance performance (Seto et al., 2013).

There were no significant differences among the female groups, with genotype frequencies of 47.8% RX, 23.8% XX, and 28.4% RR (*p* > 0.05). On the other hand, the female rowing athletes in this study mainly carried the 577 R allele, and previous research indicated that the frequency of the 577 R allele on top-level Polish rowers was higher (Jastrzębski et al., 2014). Shang *et al.* (2010) reported that the 577X allele of Chinese endurance female athletes was overrepresented and significantly different compared with that of the controls (51.3 *vs* 41.1%, *p* = 0.019). The 577 R allele was significantly higher in elite speed and power athletes than in the control group (Cięszczyk et al., 2011; Papadimitriou et al., 2016; Weyerstraß et al., 2018). The reason may be that rowing is not strictly an endurance sport with a mixed character of endurance, isokinetic strength, and power (Ahmetov et al., 2010). The 577 R allele has been demonstrated to be related to muscle mass (Galeandro et al., 2017). A meta-analysis has clearly summarized the associations between the RX and RR genotypes and the 577 R allele with power-oriented performance (Stucky-Byler et al., 2018; Lammi et al., 2019). Furthermore, the RR genotype might contribute to generating powerful and forceful muscle contractions (Del Coso et al., 2019). However, in our study, the trait variables of female athletes with *ACTN3* genotypes had no significant linear regression (*p* > 0.05). A previous study found that there were significant differences in the peak heart rate of the RR genotype and RX genotype in the male group but no significant differences in the female group, and high values of all endurance indexes appeared in the female group with the XX homozygous genotype (Deschamps et al., 2015). Similarly, in this study, the 577X allele was not significantly associated with any traits of female athletes, consistent with the results of excellent endurance-oriented athletes such as Italian rowers (Paparini et al., 2007), Russian rowers (Weyerstraß et al., 2018), and Hungarian rowers (Bosnyák et al., 2015). This could be explained by the fact that rowing is a complex discipline with a vigorous power-start demanding starting anaerobic capacity, an immediately high aerobic steady state, and an extremely exhausting spurt.

## Conclusion

As conserved genes, *ACE* and *ACTN3* polymorphisms have been reported in endurance, explosive power, sensitivity, sports injury training, and other related studies, and the results are consistent or inconsistent with each other, whether in outstanding athletes or in the normal population. Athletes are people with outstanding performance phenotypes. The sports ability phenotype is a complex phenotypic trait that is not regulated by a single gene polymorphism; therefore, good rowing athletes also do not have a single *ACE* or *ACTN3* genotype, which may be advantageous. These results suggest that the *ACE* I allele and XX genotype might genetically affect the endurance traits of male athletes, with the RR genotype being a power trait. The *ACE* D allele might genetically affect the strength traits of female athletes. Therefore, the influence of genes on the performance of movement is extensive, multifactorial, and universal. However, this is the first study to compare the gene contribution with the quantitative traits of rowing athletes. This study is also limited by the fine genetic structure of the Chinese Han population, the sample size of the athletes, the discipline of rowing, regional differences, sports performance-related traits, and other factors. These limitations will be gradually addressed in our future research.

## Data Availability

The raw data supporting the conclusion of this article will be made available by the authors, without undue reservation.

## Abbreviations

FVC, forced vital capacity; VO2max, maximal oxygen uptake; PBP, prone bench pull; LBS, loaded barbell squat; PCR, polymerase chain reaction; ANOVA, analysis of variance; SNP, single-nucleotide polymorphism; ACE, angiotensin-converting enzyme; ACTN3, α-actinin-3; STREGA, Strengthening the Reporting of Genetic Association Studies; HWE, Hardy–Weinberg equilibrium; ALFA, NCBI Allele Frequency Aggregator; gnomAD, Genome Aggregation database; 1KGP, 1,000 Genomes Project; ChinaMAP, China Metabolic Analytics Project; CHB, Han Chinese in Beijing; CHS, Southern Han Chinese; Y DNA, Y chromosome; mtDNA, mitochondrial deoxyribonucleic acid.
